# Coronary computed tomography versus coronary angiography for preoperative coronary assessment before valve surgery

**DOI:** 10.1186/s43044-021-00180-7

**Published:** 2021-07-05

**Authors:** Abdalla Elagha, Waleed Khaled, Sahar Gamal, Mohamed Helmy, Ayman Kaddah

**Affiliations:** 1 Cardiovascular Department, Kasr-Alainy Hospital, Cairo University, 1 Saraya St., Third Floor, Manial, Cairo, Egypt; 2 Cardiovascular Department, National Heart Institute, Cairo, Egypt

**Keywords:** Coronary artery disease (CAD), Coronary computed tomography angiography (CCTA), Coronary angiography (CAG), Preoperative coronary assessment

## Abstract

**Background:**

Conventional coronary angiography (CAG) is currently the gold standard technique for the assessment of coronary arteries prior to cardiac valve surgery. Although CAG is a relatively safe procedure, however, it is still an invasive procedure, and it has potential hazards and complications. Coronary computed tomography angiography (CCTA) is a non-invasive technique that has emerged robustly as an excellent and attractive tool for delineating coronary anatomy. Therefore, we sought to evaluate the accuracy of CCTA when compared with the gold standard CAG in the evaluation of coronary arteries before valve surgery. We screened 111 consecutive patients with VHD undergoing a routine cardiac catheterization for preoperative evaluation of CAD. Fifty patients were eligible and underwent both CAG and CCTA. Significant coronary stenosis was defined as a luminal diameter decrease of ≥ 50%. Additionally, ectasia, calcifications, and congenital coronary anomalies were analyzed. Also, we compared both techniques regarding radiation dose, contrast volume, and complications. Non-evaluable segments were excluded from all levels of analysis. Sixty-one patients were excluded from the study due to various reasons.

**Results:**

Among the 50 patients of the study population, 27 (54%) were males. The prevalence of significant CAD in the study population was 19.6% according to the patient-based analysis, and CAG could have been avoided in 80.4% of patients with a true-negative CCTA result. Diagnostic accuracy of CCTA for detection of significant stenosis was evaluated regarding sensitivity and specificity, positive predictive value, negative predictive value, and overall accuracy of CCTA, which was 87.5%, 99.6%,87.5%, 99.6%, and 99.2%, respectively, for segmental-based analysis; 86%, 100%, 100%, 99%, and 99%, respectively, for vessel-based analysis; and 77.8%,100%,100%, 94.9%, and 95.7%, respectively, for patient-based analysis. Fewer rates of complications were encountered with CCTA. Additional information obtained like calcifications and congenital anomalies was diagnosed better with CCTA than CAG.

**Conclusion:**

Owing mainly to its high negative predictive value, a well-performed CCTA exam is an excellent method to rule out coronary artery disease, specially in patients who are not at high risk of atherosclerosis.

## Background

Preoperative coronary angiography (CAG) for the assessment of concomitant significant coronary artery stenosis is a well-established standard procedure [1, 2]. Although coronary angiography is a relatively safe procedure, however, it is still an invasive procedure and has potential hazards, specially in patients with CAD and co-morbidities [3]. Furthermore, invasive coronary angiography usually requires a short hospital stay and causes patient discomfort. During the last decade till now, coronary computed tomography (CCTA) has emerged and proved its excellent utility in the assessment of CAD [4]. In addition to being a non-invasive tool, it has shown high negative predictive value in ruling out obstructive CAD [5].

Therefore, we sought to evaluate the accuracy of coronary computed tomography angiography when compared with the gold standard coronary angiography in the evaluation of coronary arteries before valve surgery.

## Methods

### Patient selection

Prospectively, we screened 111 consecutive patients with valvular heart disease undergoing a routine cardiac catheterization for preoperative evaluation of CAD, during a 26-month period (December 2016 to January 2019). Sixty-one patients were excluded from the study due to various reasons (refused to provide written informed consent, renal impairment, pregnancy, acute heart failure, limited time to perform both studies before surgery, improper heart rate, and rhythm for CCTA examination). The final population was 50 patients, who provided written informed consent, had no history of CAD, and were hemodynamically stable before both tests.

### Patient preparation

Both CCTA and CAG examinations were performed within a 1-week duration. The first test performed was CAG in 96% of patients. One hour prior to CCTA, heart rate control was attempted in every patient—if the baseline heart rate was > 65 bpm—using an oral beta blocker (2.5–10 mg bisoprolol). Patients with uncontrolled heart rates needed an additional bolus of intravenous propranolol (1–2 mg).

Before the scan, all patients received 0.5 mg of sublingual nitroglycerin. Both CCTA and CAG data were evaluated by operators blinded to the results of the other test.

### Conventional coronary angiography

All CAG procedures were performed by two experienced cardiologists (> 10 years’ experience in the cath lab). Vascular access was obtained through a femoral or radial approach.

Coronary artery segments reported to contain a stenotic lesion (or occlusion) of ≥ 50% were evaluated by quantitative coronary angiography (QCA) in two orthogonal views. Segments distal to a totally occluded vessel were excluded from the analysis. All other segments were included for the evaluation of the agreement with CCTA. Each coronary artery segment was classified as normal, atherosclerotic (with no significant stenosis < 50% luminal narrowing), stenotic (≥ 50% luminal narrowing), or absent (not visualized or non-existing branch).

#### Multislice computed tomography

All patients had CCTA conducted using a dual-source 64 CT system (Somatom-Definition, Siemens).

The study included a pre-scan calcium scoring, followed by a contrast-enhanced scan. All patients received non-ionic, low-osmolar contrast. The entire scan was ECG-gated used to retrospectively reconstruct datasets at the mid-to-late diastolic phase of each cardiac cycle (75%).

Using the results of selective coronary angiography as the gold standard, the evaluation was performed on a per-segment, per-vessel, and per-patient basis. Data obtained from CCTA were evaluated using trans-axial images, as well as other reconstruction modalities: multiplanar reconstructions (MPR), maximal intensity projection (MIP), and curved MPR. Coronary segments were described according to the “Braunwald coronary anatomic model” that classify coronary segments into an 18-segment model, and it was used to evaluate coronary tree by both methods. All segments—evaluated by both CCTA and CAG—were labeled either as “significant” stenosis (≥ 50% luminal narrowing) or “insignificant” stenosis (< 50% luminal narrowing).

### Statistical analysis

Data were analyzed using MedCalc© version-15.8 (MedCalc© Software, Belgium). Numerical variables were presented as mean ± SD (range) and categorical variables as number and percentage. Paired numerical data were compared using paired samples t-test and paired categorical data using the McNemar test. The diagnostic accuracy of CCTA was compared with coronary angiography using 2 × 2 contingency tables to calculate the sensitivity, specificity, positive and negative predictive values, and overall accuracy for each segment, each vessel separately, and each patient.

## Results

The studied population includes 23 (46%) females and 27 (54%) males. The mean age was 51 ± 7.7 years. The demographic characteristics are presented in Table 1.

**Table 1:** Patient demographics: characteristics of patients with a prevalence of CAD by CCTA and CAG with each risk factor

Risk factor		N (%)	Prevalence of CAD by CCTA, N (%)	Prevalence of CAD by CAG
Diabetes		8 (16%)	5 (10%)	5 (10%)
Hypertension		19 (38%)	14 (28%)	1 (26%)
Smoking	Ex-smoker	10 (20%)	8 (16%)	7 (14%)
	Still smoking	10 (20%)	2 (4%)	7 (14%)
Dyslipidemia		2 (4%)	7 (14%)	2 (4%)
Valve procedure (balloon)		1 (2%)	0	0
Stroke		1 (2%)	1 (2%)	1 (2%)
TIA		4 (8%)	1 (2%)	1 (2%)
PVD		1 (2%)	1 (2%)	1 (2%)
On regular medical treatment	Anti-ischemic	14 (28%)	12 (24%)	10 (20%)
	Antifailure	2 (4%)	1 (2%)	1 (2%)
	Both	4 (8%)	2 (4%)	2 (4%)
Positive family history		11 (22%)	10 (20%)	10 (20%)
Allergic (skin, drug)		4 (8%)	2 (4%)	2 (4%)
Asthmatic		5 (10%)	4 (8%)	4 (8%)

The average heart rate during the CCTA scan was 67.5 ± 17.7 beats/min (range 55–79). The average scan time of the CCTA scan was significantly shorter (12 ± 3 s) when compared with 839 ± 285 s as the total fluoroscopic time during diagnostic CAG.

In this study, there were 8 (16%) patients who encountered complication from CCTA scan; one patient (2%) had transient renal impairment, 3 patients (6%) had dyspnea, 4 patients (8%) had contrast allergy, and 4 patients (8%) had flushing. On the other hand, 13 patients (26%) had complications from CAG; 2% had vasovagal reaction, 8% had contrast allergy, and 18% suffered local hematoma at vascular access. The average contrast volume of CCTA was 85.4 ± 6.1 ml, which was lower compared with 95.4 ± 31.7 ml at CAG. The average total radiation dose for CCTA was 1115 ± 496.5 mGy, which was comparable to 1112.7 ± 413.9 mGy at CAG.

### Evaluation of stenosis by CCTA and CAG

Regarding per-segment analyses, a total of 900 coronary segments were subjected to evaluation by both techniques. Among those 900 segments, 136 segments were anatomically absent (segment 15 “left-PDA” being the most absent). When comparing the results of CCTA with invasive angiography, a total net of 764 segments were suitable for evaluation and distributed as seen in Table 2.

**Table 2:** Distribution of normal, non-stenotic, significantly stenotic, and non-evaluable segments among both techniques

Segment evaluation		CCTA		CAG	
		Number	Percentage (%)	Number	Percentage (%)
No significant stenosis	Normal segments	513	67.1%	529	69.2%
	< 50 lesion	226	29.6%	168	22%
Significant stenosis	≥ 50% lesion	23	3%	21	2.7%
	Totally occluded	2	0.3%	3	0.4%
Unevaluable		**0**	**0%**	43	5.7%
Total		764	100%	764	100%

Non-evaluable segments in coronary angiography were 43 segments (6% of total segments). The cause of being non-evaluable was the difficult engagement of the left coronary ostium: either due to aortic-root dilatation in most cases or aortic coarctation in one case. In contrast, there was no non-evaluable segment by CCTA; this was attributed in our study to low calcium score, proper CCTA techniques, and appropriate patient preparation.

**Fig. 1:**
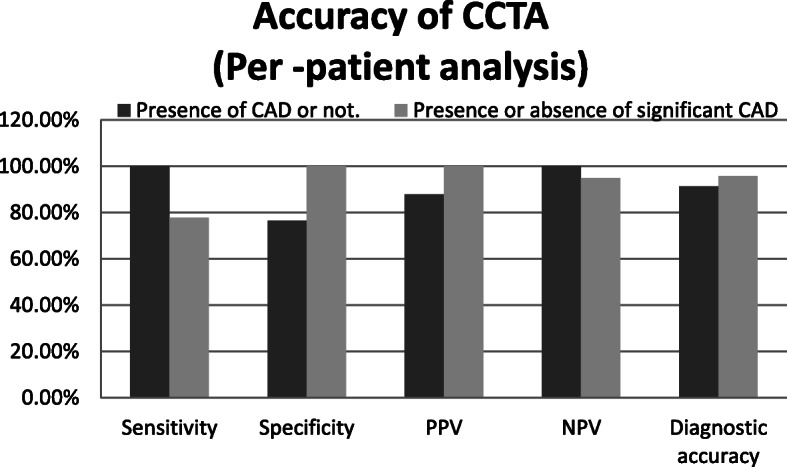
Column graph demonstrates the accuracy of CCTA for the diagnosis of the presence of both CAD and significant CAD

In a segment-based analysis (for identifying significant coronary lesion (stenosis ≥ 50%)), sensitivity, specificity, PPV, and NPV of CCTA were 87.5%, 99.6%, 87.5%, and 99.6% respectively, with a diagnostic accuracy of 99.2% (Fig. 1).

Regarding vessel-based analyses, only 190/200 vessels were evaluable, while 10 vessels contained non-evaluable segments and were excluded from further analysis. Logically, segments of one vessel branch were combined. The coronary tree was classified into the left main trunk (LMT), left anterior descending artery (LAD), left circumflex artery (LCX), and right coronary artery (RCA). The vessels with only a single segment interpretable were excluded from the vessel-based analysis. The distribution of normal and non-significantly stenotic vessels versus significantly stenotic and totally occluded vessels among both techniques is illustrated in Table 3.

**Table 3:** Distribution of various degrees of CAD per-vessel analysis among both techniques

	CCTA		CAG	
	Normal or no significant stenosis	Significant stenosis or totally occluded	Normal or no significant stenosis	Significant stenosis or totally occluded
RCA	46 (92%)	4 (8%)	46 (92%)	4 (8%)
LMT	47 (100%)	0 (0%)	47 (100%)	0 (0%)
LAD	**41 (89.1%)**	**5 (10.9%)**	**39 (84.8%)**	**7 (15.2%)**
LCX	44 (93.6%)	3 (6.4%)	44 (93.6%)	3 (6.4%)

From the previous table, we concluded that assessment of LMT, RCA, and LCX in this study was performed accurately; CCTA could rule out successfully significant lesion at LMT and detected four significant RCA lesions and three significant LCX lesions. In other words, CCTA could exclude with confidence the presence of LMT, RCA, and LCX significant stenosis among the rest of the patients with 100% diagnostic accuracy. Although there was a little decrease in diagnostic accuracy of CCTA compared to CAG regarding the diagnosis of significant stenosis in LAD vessel, however, it reaches 95.7%, which still represents a high level of diagnostic accuracy as seen in Table 3. However, when accuracy was calculated for all vessels without discretion, overall sensitivity, specificity, PPV, and NPV were 86%, 100%, 100%, and 99%, respectively, with an overall excellent diagnostic accuracy of 99% as seen in Table 4.

**Table 4:** Accuracy of CCTA for the diagnosis of significant stenosis in each (and overall) coronary artery per-vessel analysis

	RCA	LM	LAD	LCX	All vessels
Sensitivity	100%	100%	**71.4%**	100.0%	86%
Specificity	100%	100%	**100.0%**	100.0%	100.0%
PPV	100%	100%	**100.0%**	100.0%	100.0%
NPV	100%	100%	**95.1%**	100.0%	99%
Diagnostic accuracy	100%	100%	**95.7%**	100.0%	99%

Regarding patient-based analyses, all patients who had vessels with unevaluable segments by coronary angiography were excluded from the assessment of diagnostic accuracy. In this study, we found 39 (84.8%) patients by CCTA who had no significant stenosis, compared to 37 (80.4%) patients by CAG. At the same time, there were only 7 (15.2%) patients by CCTA who had significant stenosis, compared to 9 (19.6%) patients by CAG. The diagnostic accuracy of CCTA for the diagnosis of significant CAD was high (compared to standard coronary angiography), which is illustrated in Fig. 1.

#### Additional findings

##### A. Evaluation of calcifications

Only one patient (2%) had a total coronary calcium score of 400 or more. The mean total coronary calcium score, according to the Agatston scoring system, was 50 ± 146 units. A total of 766 coronary segments were evaluated for calcification; 739 (96.5%) segments by CCTA versus 718 (93.6%) segments by CAG were free of calcification. At the same time, 27 (3.5%) segments by CCTA versus 4 (0.5%) segments by CAG showed calcifications of variable degree (there were 44 segments which were unevaluable by CAG), and this difference was statistically significant (p = 0.01).

##### B. Evaluation of ectasia

There were 45 (5.9%) segments by CCTA versus 30 (3.9%) segments by CAG that showed various degrees of coronary ectasia. However, this difference was statistically insignificant (p = 0.06).

##### C. Evaluation of congenital anomalies

A total of 200 coronary vessels were evaluated for the presence or absence of congenital anomalies regarding the origin and course. There were 11 (5.5%) vessels by CCTA versus 3 (1.5%) vessels by CAG that showed anomalous origin or course, and this difference was statistically significant (p = 0.03).

##### D. Findings pertinent to CCTA only

There were 11 patients who had dilated ascending aorta ranging from 4.1 to 7.4 cm. Also, one patient had LV apical thrombus, and another patient had consolidated patches of pneumonia. Lastly, one patient had encysted pleural effusion.

## Discussion

Our current prospective study was conducted to answer some frequently asked questions: “Do we really need an invasive preoperative coronary angiography study prior to every single valve surgery in eligible patient?” and “Is there any other available non-invasive test that can accomplish this task accurately without taking the patient to the cath lab?”

The main finding of our study is that CCTA has high diagnostic accuracy and therefore can rule out significant CAD in most of the studied patients with valvular heart disease. Moreover, it can provide more accurate data about the anatomy of the coronary arteries and its wall’s pathology like calcifications, and in some cases, it is the only possible accurate imaging tool that could visualize the coronaries in some situations like in cases with hugely dilated aortic root and aortic coarctation.

The published ACC/AHA guidelines 2014 stated that CCTA is reasonable to exclude the presence of significant obstructive CAD in selected patients with a low/intermediate pretest probability of CAD, while a positive CCTA is to be confirmed with invasive CAG [1]. Also, after we have already started our current study, ESC guidelines of VHD (published in 2017) stated that CT angiography should be considered as an alternative to CAG before valve surgery in patients with severe VHD and low probability of CAD or in whom CAG is technically not feasible or associated with a high risk [6]. Actually, performing CCTA—with optimal preparation—in such a patient and then sending him/her directly to valve surgery bypassing CAG could be feasible.

Our results showed a high degree of accuracy of how CCTA could clinch the presence of CAD and significant coronary stenosis (≥ 50%). Per-segment analysis of data obtained showed very high specificity, NPV, and overall accuracy of CCTA in the assessment of coronary obstruction. These results were important for the exclusion of significant CAD before surgery. Our findings were consistent with several previously published reports like Hoffmann et al. who studied the diagnostic accuracy of 16-slice CCTA compared with invasive CAG in 1384 consecutive patients primarily with suspected CAD and concluded that sensitivity, specificity, PPV, and NPV of per-segment analysis of CCTA were 95%, 98%, 87%, and 99%, respectively, which is nearly similar to our study results [7]. Also, Reant et al. studied 40 consecutive patients with severe acquired valvular disease scheduled for preoperative invasive CAG, where diagnostic accuracy of 16-slice CCTA in the per-segment analysis was as follows: sensitivity 77.7%, specificity 98%, PPV 42.4%, and NPV 99%. They reported that the main cause of false-positive or false-negative results or non-assessable evaluations was severe coronary calcification [8].

Another systematic review made by d’Othee et al. about the diagnostic accuracy of CCTA includes 50 patients, and they reported similar results to ours, with a high sensitivity and specificity of 64-CCTA for detecting significant coronary stenosis (91% and 92%, respectively) and a very good overall PPV of 80% and a NPV of 97% [9]. A larger study that included 452 consecutive patients undergoing routine preoperative CAG revealed that for segment-based analysis of 64-slice CCTA, overall sensitivity, specificity, PPV, and NPV were 89%, 97%, 38%, and 100%, respectively. They attributed marked reduction in PPV in this study was due to including all non-evaluable segments and considering them as positive for significant disease [10].

So, according to our study, CCTA had a high specificity, NPV, and overall accuracy with very good sensitivity and PPV to rule out significant CAD on a segment-based analysis. These results were reached by including only evaluable coronary segments in the statistical analysis. However, if non-evaluable segments were included as in some previously mentioned studies, a number of false-positive segments would increase and the diagnostic accuracy of CCTA would be affected, mainly affecting the specificity and PPV, as shown in some previously mentioned studies [7–10]. Also, it was mentioned that calcification was mainly responsible for the false-positive and false-negative results in a previous study [8]. Fortunately, most of our patient has no or little coronary calcification (only one patient had a calcium score > 400). This is attributed mainly to the relatively young age of the studied population and also to the fact that most of the cases of VHD scheduled for surgery in our institution and country are related to rheumatic heart disease, not age-related valvular degeneration, which is usually associated with heavy calcifications.

A per-vessel analysis is usually more important than per-segment analysis; because a single distal segment stenosis usually will not have a significant impact on the management of such patients.

For vessel-based analysis, the diagnostic accuracy of CCTA in our study was 100% for all vessels except LAD (95.7%) due to missing two significantly diseased vessels at LAD territory. Anyhow, these two LAD vessels were diseased distally, and missing them would not affect the overall strategy of management. Actually, CCTA detected successfully four significant RCA lesions and three significant LCX lesions. Also, it excluded the presence of any left main significant lesion with 100% diagnostic accuracy.

It is obvious that the high diagnostic accuracy of CCTA is noticed for all vessels except distal LAD in 2 cases, due to the very small caliber of the vessel distally. Actually, these 2 vessels affected markedly the sensitivity and NPV per-LAD analysis (71.4% and 95.1%, respectively). As well known, this can be explained statistically by the fact that these two “false-negative” results affect the mathematical calculation of the sensitivity and negative predictive value.

These study results are consistent with previous studies showing a fairly high diagnostic accuracy of CCTA on each vessel-based assessment. One example was demonstrated by Pouleur et al. who evaluated the diagnostic accuracy of 40-slice multidetector CT to detect coronary disease in patients prior to cardiac valve surgery. The per-vessel analysis for 82 patients by CCTA showed significantly higher accuracy for the left main (99%) followed by RCA (93%) then LCX (91%) while the lower accuracy was in LAD (88%), which nearly agrees with our results [11]. Also, Bettencourt et al. studied the diagnostic accuracy of 64-MDCT for the detection of significant stenosis on 452 consecutive patients with VHD and revealed that on the vessel-based analysis, the overall sensitivity, specificity, PPV, and NPV were 90%, 92%, 48%, and 99%, respectively, with an overall diagnostic accuracy of 92% for CCTA compared to the CAG results [10]. Other researchers showed that on a vessel-based analysis, the overall sensitivity was 89.6%, specificity was 97.7%, PPV was 88.9%, and NPV was 97.6% [12].

Actually, the previous studies and others [13] agree with our results except for PPV, which was lower in those studies than in our study. Simply, it is explained by the inclusion of non-evaluable segment in their studies and considering them positive for significant stenosis which increases the false-positive results and consequently decreases the PPV.

However, the finding of interest is the persistently high NPV in our and other studies, which was not influenced by including the non-evaluable segment in the analysis. This is of great value as this ensures the value of the CCTA as a rule-out modality of significant coronary stenosis. This concept is consistent with the findings addressed by Abdulla et al. who conducted a meta-analysis including 27 studies and reported the negative effect of these non-evaluable segments on the specificity and PPV of CCTA [14].

For patient-based analysis, there were 39 (84.8%) patients by CCTA versus 37 (80.4%) patients by CAG who had no significant stenosis. Table 5 illustrates the different previous study results compared to our study results regarding the per-patient analysis level [7, 10–13, 16, 17]. Again, the relative decrease of sensitivity (77.8%) and NPV (94.9%) in our study is explained by the slightly increased false-negative results (distal LAD lesions).

**Table 5:** The different study results of CCTA accuracy for the detection of significant coronary lesions ≥ 50% compared to our study results regarding per-patient analysis level

Studies	Pt (n)	Detector (n)	Sensit.	Specif.	PPV	NPV
Our study	**50**	**64**	**77.8**	**100%**	**100%**	**94.9%**
Scheffel et al., 2007 [12]	50	64	100%	95%	87%	100%
Bettencourt et al., 2009 [10]	452	64	95%	89%	66%	99%
Larsen et al., [15]	181	64–320	78%	81%	81%	83%
Opolski et al., 2016 [17]	1107	< 64	93%	89%	–	–
Joshi et al. 2016 [16]	50	128	100%	91.3%	50%	100%
Hoffmann et al., 2005 [7]	103	16	95%	97%	98%	94%
Meijboom et al., 2006 [13]	145	64	100%	92%	82%	100%
Pouleur et al., 2007 [11]	82	40	93%	90%	55%	99%

Also, we noticed from the revision of most of the previous studies that the specificity and PPV were mostly affected and were lower than our study. It is explained by our exclusion of the non-evaluable segments, while the other studies include them and considered them positive, which increase the false-positive results [7–10, 16].

**Fig. 2:**
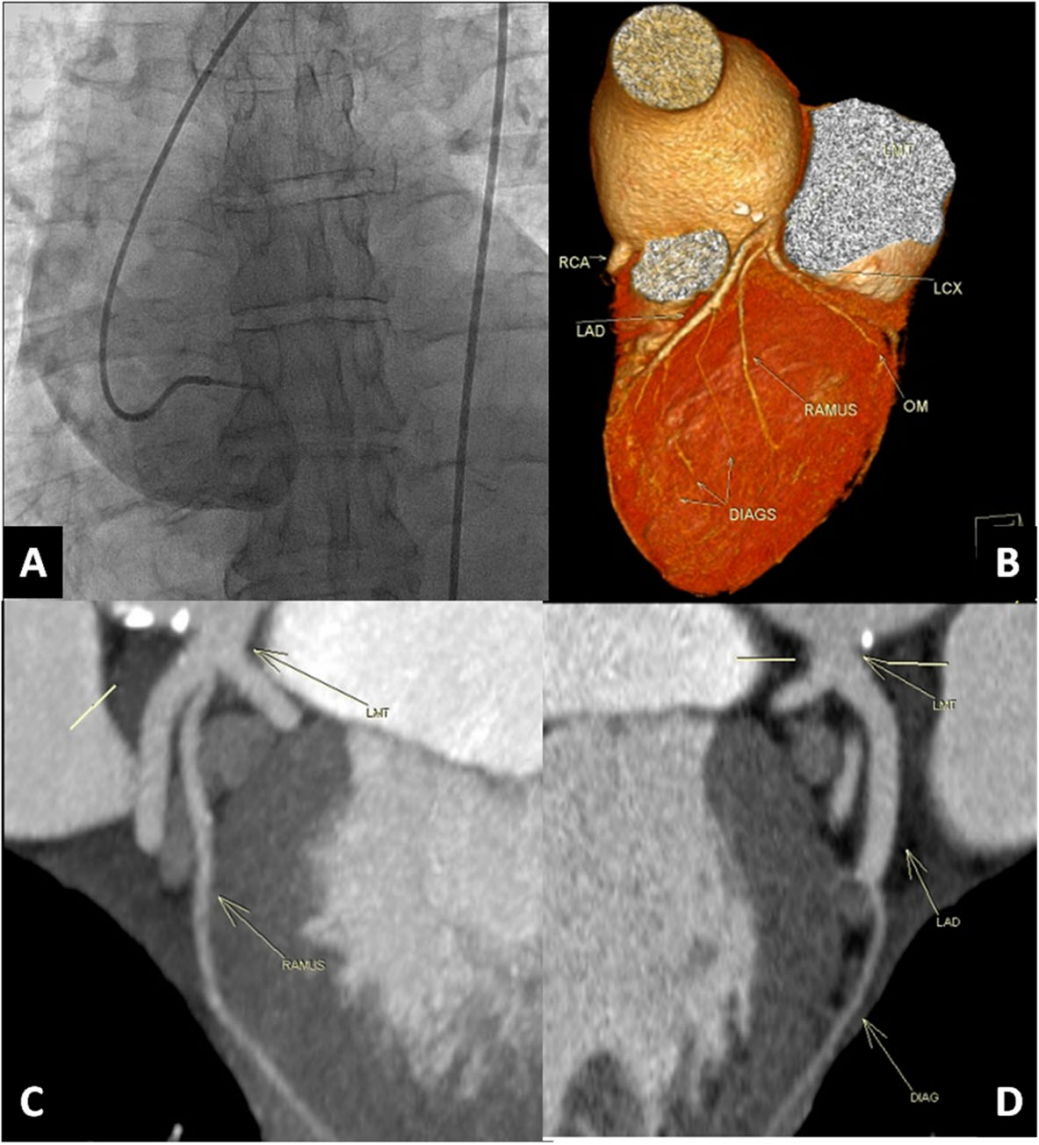
Demonstration of a case of severe aortic regurgitation with dilated aortic root. **A** Non-selective injection of contrast by catheter due to the difficult engagement of the left main trunk. **B** Volume-rendering technique (VRT) by CCTA shows the anatomy of different coronary arteries. **C**, **D** Multiplanar reconstruction (MPR) of CCTA reveals the normal LAD, ramus, LCX, and diagonal arteries

**Fig. 3:**
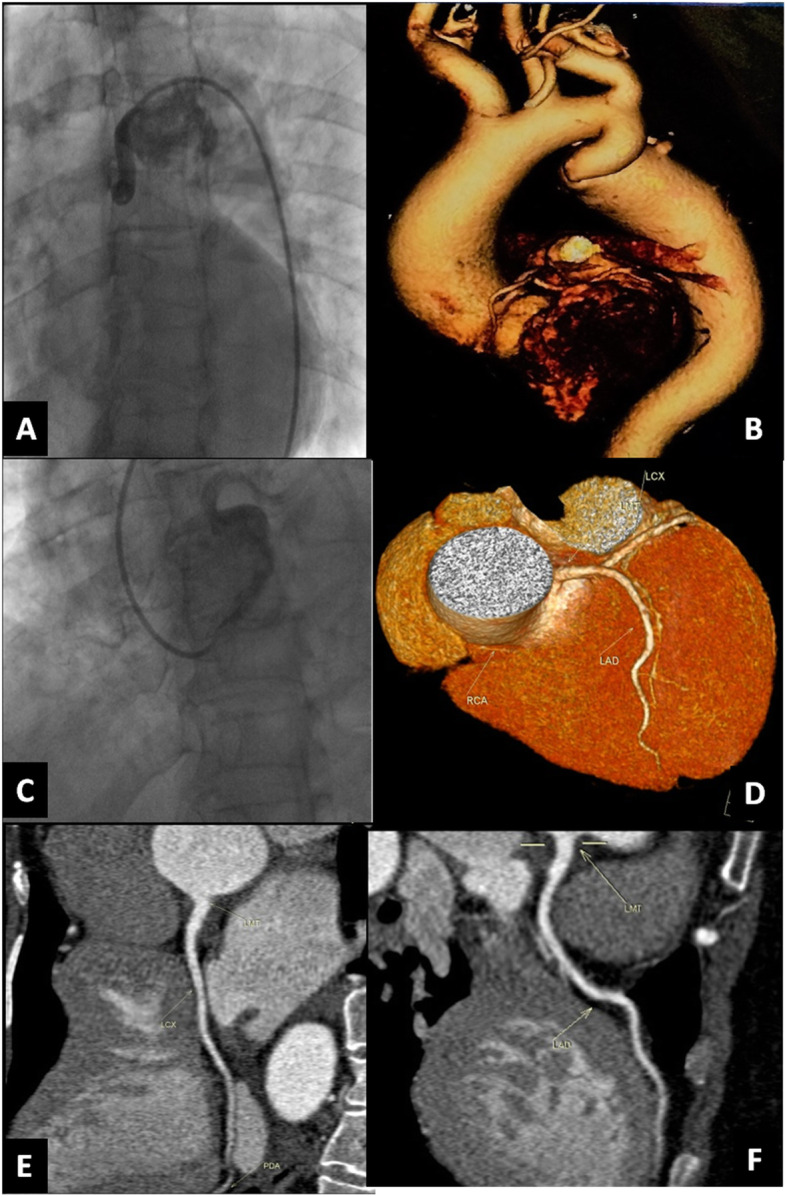
Demonstration of a case of aortic coarctation with the inability to pass the catheter. **A** Difficult passage of catheter due to aortic coarctation. **B** VRT of CT aortography reveals aortic coarctation just distal to the origin of the left subclavian artery. **C** Non-selective injection of contrast by catheter due to the difficult engagement of coronaries. **D** VRT of CCTA reveals the anatomy of different coronary arteries. **E**, **F** MPR of CCTA reveals normal LCX and LAD, respectively

The non-evaluable segments in our study were not at CCTA but at coronary angiography due to the difficult catheter engagement of the left coronary artery, either due to aortic root dilatation or aortic coarctation. Figures 2 and 3 are good examples of these situations. Alternatively, the non-evaluable segments in other studies were in CCTA due to poor image quality caused by motion or blooming artifacts [8].

Although CT coronary angiography for preoperative evaluation in VHD is increasingly being used with high accuracy for the detection of significant coronary stenosis, there is slightly lower diagnostic yield in cases of aortic stenosis (AS) due to the frequent aortic and coronary calcifications [17]. A word worth mentioning is that cardiac CT has had a major emergence in the field of preoperative assessment of transcatheter aortic valve replacement (TAVR), as it is crucial in the assessment of the annular area, diameter, valve leaflet morphology and calcification, and peripheral vascular assessment [18, 19]. The severity of the aortic valve “Agatston” calcium score—calculated by cardiac CT—has been shown to correlate with the degree of paravalvular leak following TAVR [20]. In the current study, we did not have any patient with aortic stenosis that was treated with TAVR, although all measurements needed by CT were measurable. Regarding our results of coronary calcification, CCTA had a superior capacity for diagnosing calcifications and its degree more than CAG, a logical result since the calcium score technique is now a well-established quantitative method.

Additional information provided by CCTA in our study was not limited to coronary calcifications but was extended to include better data for the detection of coronary artery anomalies. CCTA is a 3D imaging modality that can view the heart in any plane, with a large field of view, which allows better visualization of the coronary anomaly.

Finally, complications were less frequent and less harmful with the CCTA technique than with CAG. This is explained by the nature of each technique, being non-invasive and quick with CCTA, but invasive and longer in time with CAG.

### Study limitations

We acknowledge that our study has several limitations. The study was conducted at two centers only (not multicenter) with experience in CCTA, and as such, results may not be representative for all centers. Limited by our resources, we could utilize only dual-source 64-detector scanners, which really accomplished the task needed; however, a higher grade CT scanner with 256 or 320 slices or even more with improved technology may actually reduce the scan time, lower radiation exposure, and improve spatial resolution. Another limit is the small number of sample size (n = 50), and of those, only patients scheduled for elective valve surgery were included. Also, there was a limited number of patients with isolated one valve lesion (only two patients) as rheumatic valvular heart disease is endemic in our region. Also, there were no patients with previous coronary bypass surgery or stents in our study. Also, the study did not include patients with rhythm disturbance (as atrial fibrillation), so accuracy in our study was slightly higher and all CCTA images were interpretable. Finally, as mentioned before, we did not include non-evaluable segments in the per-segment and per-vessel analysis as done in previous similar studies.

## Conclusion

Owing to its high negative predictive value, CCTA is useful to rule out CAD in the majority of patients. The potential role of CCTA proposed here is not to replace cardiac catheterization as the gold standard preoperative tool in CAD assessment, but rather a trial to avoid taking every single eligible patient to the invasive cath lab before sending him/her next to the surgical theater. Consequently, our findings support the potential for the reduction of unnecessary downstream testing and catheter-related complications and costs. By relevance, a normal CCTA examination confers an excellent prognosis, thus supporting its clinical utility if used preoperatively in patients with suspected CAD and planned for valve surgery. We believe more studies are needed to understand better which populations would be fitting for coronary CTA to assess the presence or absence of CAD.

## Data Availability

All data generated or analyzed during this study are included in this published article [and its supplementary information files].

## Abbreviations

CAG: Conventional coronary angiography
CCTA: Coronary computed tomography angiography
CAD: Coronary artery disease
QCA: Quantitative coronary angiography
MPR: Multiplanar reconstructions
MIP: Maximal intensity projection
